# Neurosteroid Transport in the Brain: Role of ABC and SLC Transporters

**DOI:** 10.3389/fphar.2018.00354

**Published:** 2018-04-11

**Authors:** Markus Grube, Paul Hagen, Gabriele Jedlitschky

**Affiliations:** Department of Pharmacology, Center of Drug Absorption and Transport, University Medicine Greifswald, Greifswald, Germany

**Keywords:** ATP-binding cassette transporters, blood–brain barrier, dehydroepiandrosterone, DHEAS, neuroactive steroids, pregnenolone sulfate, solute carriers

## Abstract

Neurosteroids, comprising pregnane, androstane, and sulfated steroids can alter neuronal excitability through interaction with ligand-gated ion channels and other receptors and have therefore a therapeutic potential in several brain disorders. They can be formed in brain cells or are synthesized by an endocrine gland and reach the brain by penetrating the blood–brain barrier (BBB). Especially sulfated steroids such as pregnenolone sulfate (PregS) and dehydroepiandrosterone sulfate (DHEAS) depend on transporter proteins to cross membranes. In this review, we discuss the involvement of ATP-binding cassette (ABC)- and solute carrier (SLC)-type membrane proteins in the transport of these compounds at the BBB and in the choroid plexus (CP), but also in the secretion from neurons and glial cells. Among the ABC transporters, especially BCRP (ABCG2) and several MRP/ABCC subfamily members (MRP1, MRP4, MRP8) are expressed in the brain and known to efflux conjugated steroids. Furthermore, several SLC transporters have been shown to mediate cellular uptake of steroid sulfates. These include members of the OATP/SLCO subfamily, namely OATP1A2 and OATP2B1, as well as OAT3 (SLC22A3), which have been reported to be expressed at the BBB, in the CP and in part in neurons. Furthermore, a role of the organic solute transporter OSTα-OSTβ (SLC51A/B) in brain DHEAS/PregS homeostasis has been proposed. This transporter was reported to be localized especially in steroidogenic cells of the cerebellum and hippocampus. To date, the impact of transporters on neurosteroid homeostasis is still poorly understood. Further insights are desirable also with regard to the therapeutic potential of these compounds.

## Introduction

Neurosteroids are cholesterol-derived compounds categorized in pregnane neurosteroids (e.g., allopregnanolone), androstane neurosteroids (e.g., androstanediol), and sulfated compounds [PregS and DHEAS] (Reddy, 2010). They can be synthesized in the central nervous system, or the compounds themselves or precursors can be taken up from the systemic circulation (Baulieu, 1997; Maninger et al., 2009). One of their main functions in the brain is to modulate excitability by interaction with membrane receptors and ion channels (Reddy, 2010). Accordingly, a therapeutic potential of these compounds or synthetic analogs has been discussed for a variety of brain disorders (Reddy, 2010). Local steroid biosynthesis in rodent and human brain has been studied since 1990s (Baulieu, 1997). Expression of key enzymes (e.g., P450 side-chain cleavage enzyme, P450c17) has been demonstrated in the principal neurons of several brain areas and in the microglia and astrocytes (reviewed in: Maninger et al., 2009; Hojo et al., 2011; Porcu et al., 2016). However, the relative contributions of the local synthesis and uptake from blood to CNS levels have still to be clarified and may vary between different neurosteroids. In contrast to the lipophilic unconjugated compounds, especially DHEAS and PregS do not diffuse across membranes at a sufficient rate due to their hydrophilic sulfate moiety. Therefore, this review focuses on DHEAS/PregS membrane transporters in the brain. Both compounds play important roles in age-related memory and learning. They can be formed from the non-sulfated precursors by SULTs with SULT2B1b as a major isoform in the human brain (Salman et al., 2011). The ratio of sulfated versus non-sulfated neurosteroids in the brain may be decisive, since sulfation can change the direction of the neuromodulating activity. While for example some non-sulfated neurosteroids such as allopregnanolone are potent positive modulators of GABA type A receptors (for review see Chisari et al., 2010; Reddy, 2010), DHEAS and PregS have been shown to antagonize the GABA effect on the GABA_A_ receptor (Seljeset et al., 2015) and to be potent allosteric agonists at NMDA receptors (Wu et al., 1991; Monnet et al., 1995). Furthermore, PregS has been shown to directly activate certain TRP channels (Harteneck, 2013).

## Dheas and Pregs Levels in Plasma, Brain, and Cerebrospinal Fluid (CSF) in Humans

**FIGURE 1:**
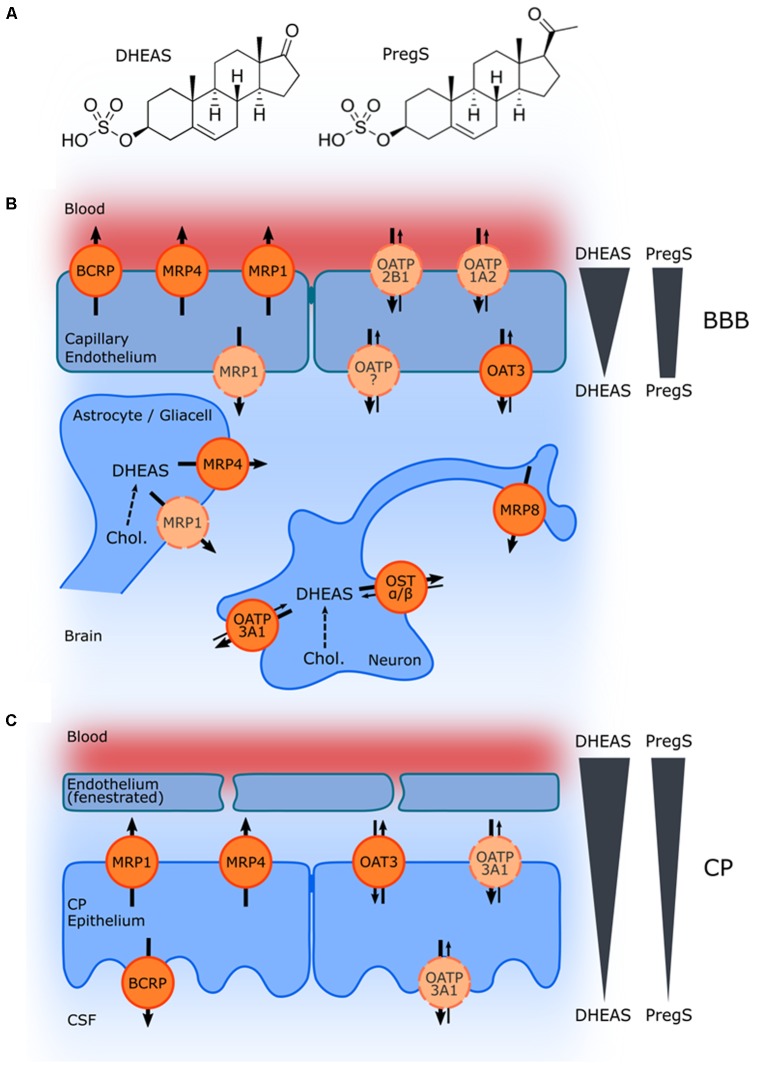
Schematic illustration of ABC and SLC transporters putatively involved in DHEAS and PregS transport and their proposed localization. **(A)** Structures of DHEAS and PregS. **(B,C)** The ABC proteins BCRP (ABCG2), MRP1, MRP4, and MRP8 (ABCC1, ABCC4, and ABCC11), and the solute carriers OAT3 (SLC22A3), OATP1A2 (SLCO1A2), OATP2B1 (SLCO2B1), and OSTα/ß (SLC51A/B) may be involved in secretion of sulfated steroids from neurons and glial cells and/or in the transport across the BBB **(B)** as well as in transport at the blood–CSF barrier in the CP **(C)**. The arrows indicate the directions of substrate transport. Proteins for which there is little or controversial evidence for expression and localization in the basal or apical membrane are indicated in light orange and by a dashed line. Concentration gradients of DHEAS and PregS across the BBB and in the CP are schematically indicated at the right side.

While the serum concentrations of the non-sulfated DHEA and pregnenolone are in the low nM range in men and women (Labrie et al., 1997; Kancheva et al., 2011), levels of DHEAS are much higher with a slight difference between men (2.3–11.5 μM) and women (1.6–6.2 μM). It should be noted that here total steroid concentrations were measured. Due to the high plasma protein binding (95% for DHEAS; Wang and Bulbrook, 1969) the concentration of the unbound steroids is accordingly lower. Generally, hormone levels decrease with age, exhibiting a maximum in young men and women (∼20 years; Labrie et al., 1997). Total PregS serum levels in adolescence range between 40 and 140 nM, and increase during pregnancy and at birth (de Peretti and Mappus, 1983; Kancheva et al., 2011). In contrast to DHEAS and PregS serum levels, less information is available on their tissue concentrations in the brain and in the CSF. In the human brain, region-dependent PregS concentrations between 5 and 40 nmol/kg have been reported with the highest levels in the striatum and hypothalamus. DHEAS concentrations were detected in a similar range with the highest levels in the striatum and cerebellum (Weill-Engerer et al., 2002). The CSF concentrations of DHEAS and PregS were much lower compared to the respective serum levels [serum/CSF ratios: 584 (Preg-conjugates), 19849 (DHEA-conjugates); Kancheva et al., 2011]. These gradients are due to the fact that both compounds cannot cross the respective barriers by passive diffusion, but depend on selective uptake and efflux proteins. ABC- and solute carrier (SLC)-type transporters may not only be relevant for the transport at the BBB and the blood–CSF barrier in the CP, but also for secretion of these compounds from neurons and glial cells (**Figure 1**).

## Transporters That May Play a Role in the Transport of Neurosteroids: ABC Transporters

ATP-binding cassette proteins can mediate a unidirectional primary active transport of a variety of compounds across membranes. Among the ABC transporters especially ABCG2, also known as the BCRP, and several ABCC/MRP subfamily members are known efflux pumps for conjugated steroids (Suzuki et al., 2003; Haimeur et al., 2004).

### BCRP/ABCG2

BCRP/ABCG2 was initially identified as a non-P-glycoprotein and non-MRP-type resistance factor from drug-selected cell lines (Doyle et al., 1998; Miyake et al., 1999). Besides anti-cancer drugs like mitoxantrone, BCRP actively transports sulfated steroids such as E_1_-3-S and DHEAS, but not unconjugated or glucuronidated steroids (Imai et al., 2003; Grube et al., 2007). The K_m_ value for E_1_-3-S calculated in isolated membrane vesicles was in the low μM range (**Table 1**). In addition, androgens such as dihydrotestosterone (DHT) have been identified as BCRP substrates (Huss et al., 2005). In human brain microvessels, ABCG2/BCRP transcript (Cooray et al., 2002; Warren et al., 2009) and protein was detected as one of the most abundant ABC transporters (Shawahna et al., 2011). Immunohistochemistry showed it primarily localized at the apical (luminal) side of the endothelial cells (Cooray et al., 2002; Aronica et al., 2005; Warren et al., 2009). In addition, it was detected in the apical membrane of the CP epithelium (Roberts et al., 2008a). Hence, BCRP could be involved in limiting the penetration of peripheral DHEAS and other steroids into the brain or facilitating the elimination of brain-derived DHEAS into blood. The apical localization in the CP, on the other hand, indicate that here BCRP is able to transport neurosteroids into the CSF.

**Table 1:** ABC and SLC transporters possibly involved in neurosteroid transport in the brain.

Transporter	CNS expression (mRNA and protein analysis)	CNS localization (immunohistochemistry)	Steroid substrates (*K*_m_ value)
**ABC Transporters**			
BCRP (ABCG2)	mRNA: - Total brain^1^ - Isolated brain microvessels (BMV)^2,3^ Protein: - BMV (H, M, Mo, R, P)^4,5,6,7,8^ - Isolated CP^9^	BBB: apical (luminal) (H, R); CP: apical (H, R)^2,10,11^	E_1_-3-S (6.8 μM)^12,13^ DHEAS^12^ DHT^14^
MRP1 (ABCC1)	mRNA: - Total brain^1^ - BMV^3^ - Isolated CP^15^ Protein: - Isolated CP (H, R)^9,16^	BBB: apical (H, B, M), Basal (abluminal) (R, M); CP: basal (H, R)^17,18,19,20^	E_1_-3-S (+GSH)^21^ DHEAS (+GSH) (5 μM)^22^ E_2_-17βG (1.5 μM)^23^
MRP4 (ABCC4)	mRNA: - Total brain^1^ - BMV^3^ - Isolated CP^15^ Protein: - BMV (H, M, Mo, R)^4,5,6,7^ - Isolated CP^9^	BBB: apical (H, M, R), Apical + basal (B); CP: basal (H, R); Astrocytes^3,11,19,20,24^	DHEAS (2 μM)^22^ E_2_-17βG (30 μM)^22,25^
MRP8 (ABCC11)	mRNA: - Total brain^1^	Neurons (axonal; cerebellum, cortex)^26^	E_1_-3-S (>150 μM)^27^ DHEAS (13–21 μM)^26,27^ E_2_-17βG (63 μM)^27^
**SLC Transporters**			
OATP1A2 (SLCO1A2)	mRNA: - Total brain^1^ Protein: - BMV (M, Mo)^5,6^	BBB: luminal; amacrine neurons^28,29,30,31^	DHEAS (7 μM)^32^ E_1_-3-S (16 μM)^31^
OATP2B1 (SLCO2B1)	mRNA: - Total brain^1^ Protein: - Brain capillary cells^31^ - BMV (M)^6^	BBB: luminal; CP (ependymal cells) (R); amacrine neurons^11,29,30,33^	E_1_-3-S (5–21 μM)^34,35^ DHEAS (9 μM)^35^ PregS^36^
OATP3A1 (SLCO3A1)	mRNA: - Total brain^1^ Protein: - Isolated CP^9^ - BMV (P)^8^	CP; neurons^37^	E_1_-3-S^38^
OAT3 (SLC22A3)	mRNA: - Total brain^1^ Protein: - Isolated CP^9^ - BMV (R)^7^	BBB: basal (R); CP: basal (H, R)^39,40,11,41^	E_1_-3-S (8.8 μM)^42^ DHEAS^42^
OSTα-OSTβ (SLC51A/B)		Neurons (cerebellum, Hippocampus; Purkinje cells)^43^	DHEAS (1.5 μM)^43^ PregS (6.9 μM)^43^

*Unless otherwise specified, the data refer to human tissue and transporter protein. B, bovine; BBB, blood–brain barrier (capillary endothelial cells); BMV, isolated brain microvessels; CP, choroid plexus; DHEAS, dehydroepiandrosterone sulfate; DHT, dihydrotestosterone; E_1_-3-S, estrone-3-sulfate; E_2_-17βG, estradiol-17 β-glucuronide; H, human; M, mouse; Mo, monkey; P, pig; R, rat; PregS, pregnenolone sulfate. ^1^Nishimura and Naito, 2005; ^2^Cooray et al., 2002; ^3^Warren et al., 2009; ^4^Shawahna et al., 2011; ^5^Ito et al., 2011; ^6^Uchida et al., 2011; ^7^Hoshi et al., 2013; ^8^Kubo et al., 2015; ^9^Uchida et al., 2015; ^10^Aronica et al., 2005; ^11^Roberts et al., 2008a; ^12^Grube et al., 2007; ^13^Imai et al., 2003; ^14^Huss et al., 2005; ^15^Niehof and Borlak, 2009; ^16^Gazzin et al., 2008; ^17^Rao et al., 1999; ^18^Wijnholds et al., 2000; ^19^Nies et al., 2004; ^20^Zhang et al., 2004; ^21^Qian et al., 2001; ^22^Zelcer et al., 2003; ^23^Jedlitschky et al., 1996; ^24^Leggas et al., 2004; ^25^Chen et al., 2001; ^26^Bortfeld et al., 2006; ^27^Chen et al., 2005; ^28^Gao et al., 2000; ^29^Bronger et al., 2005; ^30^Gao et al., 2015; ^31^Lee et al., 2005; ^32^Kullak-Ublick et al., 1998; ^33^Ji et al., 2012; ^34^Pizzagalli et al., 2003; ^35^Hagenbuch and Stieger, 2013; ^36^Grube et al., 2006; ^37^Huber et al., 2007; ^38^Tamai et al., 2000; ^39^Alebouyeh et al., 2003; ^40^Kikuchi et al., 2003; ^41^Mori et al., 2003; ^42^Burckhardt, 2012; ^43^Fang et al., 2010.*

### Members of the MRP/ABCC Family

Anionic conjugates of lipophilic compounds are typical substrates for several members of the MRP family. As the founding member, MRP1 (ABCC1) was identified as second export pump conferring a multidrug resistance phenotype besides the MDR1/P-glycoprotein (ABCB1) (Cole et al., 1992). MRP1 was subsequently shown to preferentially transport amphiphilic anions, especially conjugates of lipophilic compounds with glutathione, glucuronate, or sulfate (Jedlitschky et al., 1996; Loe et al., 1996). MRP1 transports in addition certain cationic or uncharged compounds, but only in co-transport with reduced glutathione (GSH; Loe et al., 1996). It also mediates the transport of E_1_-3-S and of DHEAS in a glutathione-dependent manner (Qian et al., 2001; Zelcer et al., 2003) and is expressed in several tissues (Haimeur et al., 2004). It is also expressed in brain microvessels (Warren et al., 2009); however, the exact localization and function has not been finally clarified. It was detected to be luminal in humans, bovine, and murine brain (Nies et al., 2004; Zhang et al., 2004), but also localization at the basolateral (abluminal) membrane was described in mice and rats (Kilic et al., 2008; Roberts et al., 2008a). Some groups did not detect MRP1 protein in human brain capillaries at all (Rao et al., 1999; Aronica et al., 2005). Because of the inconsistent and low-level expression, the relevance of this transporter at the BBB is so far unclear. It may play a more important role in the CP. Here, MRP1 mRNA was detected in rat and human tissue (Gazzin et al., 2008; Niehof and Borlak, 2009) and the protein was localized at the basolateral membrane (Rao et al., 1999; Wijnholds et al., 2000; Gazzin et al., 2008). Thus, it may be involved in the efflux of conjugated steroids from the CSF into blood. In addition, MRP1 mRNA was detected in cultured rat and human astrocytes (Hirrlinger et al., 2001; Spiegl-Kreinecker et al., 2002). However, the protein could not been detected in human glial cells or neurons in immunohistochemical studies (Nies et al., 2004; Aronica et al., 2005). MRP1 and MRP2 (ABCC2) share a similar substrate spectrum, but MRP2 is mainly expressed in polarized epithelial cells (Keppler, 2011). Immunostaining at the apical membrane in brain capillaries was described in rats (Miller et al., 2000), but was not observed in human and bovine brain (Nies et al., 2004; Zhang et al., 2004). Similarly, MRP3 (ABCC3) protein was not detected in human brain (Nies et al., 2004).

A more relevant efflux transporter for conjugated steroids in the brain may be MRP4/ABCC4, which exhibits a unique broad substrate specificity. MRP4 shows the remarkable capacity to transport cyclic nucleotides and MRP4 has been established as an independent regulator of intracellular cAMP levels in several cell types (Ritter et al., 2005; Jedlitschky et al., 2012; Belleville-Rolland et al., 2016). Furthermore, MRP4 transports lipid mediators such as prostanoids and conjugated steroids. DHEAS is transported by MRP4 in a glutathione-independent manner and with high affinity (K_m_ of 2 μM; Zelcer et al., 2003). It is expressed in several tissues especially in the prostate, kidney, blood cells, and brain (Kool et al., 1997; Haimeur et al., 2004; Nishimura and Naito, 2005; Niehof and Borlak, 2009; Warren et al., 2009; Shawahna et al., 2011). Here, it was localized apically in human, rodent and bovine capillaries (Nies et al., 2004; Leggas et al., 2004; Zhang et al., 2004; Roberts et al., 2008a). An additional detection at the basolateral membrane was only described in bovine brain (Zhang et al., 2004). MRP4 expression was also detected in human CP (Niehof and Borlak, 2009; Uchida et al., 2015) and it was localized to the basolateral membrane in human and murine tissue (Leggas et al., 2004). Moreover, it is expressed in glial cells. Immunofluorescence studies in the human brain revealed staining mainly in astrocytes of the subcortical white matter (Nies et al., 2004). Since glial cells are able to synthesize neurosteroids, MRP4 may account for the efflux of DHEAS and other neurosteroids from these cells for a paracrine action. Astrocytes play a critical role for the development and function of neurons and these cells in turn are regulated by steroid hormones as progesterone and DHEA (Acaz-Fonseca et al., 2016; Arbo et al., 2016). At the BBB and the CP, MRP4 may contribute to the transport of sulfated steroids from brain and CSF into blood.

A further member of the MRP family, the MRP8 (ABCC11) may be relevant with respect to neurosteroid transport. MRP8 was shown to transport DHEAS in isolated membrane vesicles with a K_m_ value of 13–21 μM, whereas the K_m_ was above 150 μM for MRP8-mediated transport of E_1_-3-S (Chen et al., 2005; Bortfeld et al., 2006). In immunofluorescence studies, it was detected preferentially in the white matter of the cortex and cerebellum and co-localized with neurofilaments indicating localization in neuronal axons (Bortfeld et al., 2006). In addition, weak immunostaining was detected in the gray matter and also in the axons of peripheral neurons. The axonal localization implies that MRP8 can mediate presynaptic efflux of neurosteroids from neurons and thus could directly participate in modulating postsynaptic neurotransmitter receptors (Bortfeld et al., 2006).

### Uptake (SLC) Transporters

Besides ABC-type efflux transporters, neurosteroid concentrations in the brain may also be modulated by uptake transporters. Since several members of the SLC superfamily have been identified as uptake transporters for steroid conjugates in general, these transporters are interesting candidates for the transport of neurosteroids. In fact, several SLCs have been shown to mediate cellular uptake of DHEAS and PregS.

### Members of the OATP/SLCO Family

Among the SLC transporters, the OATP (SLCO) family is probably the most interesting one in this context. In humans, 11 SLCO transporters exist, organized in six families (Hagenbuch and Stieger, 2013). The physiological substrate profile of the OATP transporters comprises a wide variety of endogenous organic anions including bile acids, bilirubin, thyroid hormones, and prostaglandins (Hagenbuch and Stieger, 2013). In addition, OATPs transport steroid hormone conjugates like E_1_-3-S (nearly all OATPs) or estradiol-17β-glucuronide (OATP1A2, OATP1B1, OATP1B3, OATP1C1, and OATP4A1) and the neurosteroid DHEAS (OATP1A2, OATP1B1, OATP1B3, and OATP2B1) (Hagenbuch and Stieger, 2013). The affinity of these transporters toward DHEAS was slightly above (OATP1B1 and OATP1B3) or in the range (OATP2B1 and OATP1A2) (**Table 1**) of the physiological plasma concentration (1.6–11.5 μM; Labrie et al., 1997) indicating an *in vivo* relevance of these findings. OATP1B1 and OATP1B3 are almost exclusively expressed in the human liver. Here, they are responsible for cellular uptake as a prerequisite for hepatic metabolism and elimination. In turn, the expression and function of these transporters affect systemic DHEAS levels as shown by enhanced DHEAS levels in monkeys and rats after treatment with the unspecific OATP inhibitor rifampicin (Watanabe et al., 2015; Nishizawa et al., 2017). Assuming a DHEAS transport/uptake from the blood into the brain/CSF (Kancheva et al., 2010), changes in DHEAS plasma concentration might indirectly influence the concentration in these compartments. Therefore, OATPs localized in the BBB and/ or the CP are of special interest. Indeed, OATP1A2, OATP1C1, OATP2B1, and OATP3A1 have been detected in these structures in humans (Kullak-Ublick et al., 1998; Pizzagalli et al., 2002; Huber et al., 2007; Roberts et al., 2008b; Ji et al., 2012). Since OATP1C1 is a thyroid hormone transporter and the function of OATP3A4 is only poorly understood, at present OATP1A2 and OATP2B1 are probably the most interesting members concerning neurosteroid transport in the brain. Both proteins are expressed in the endothelial cells of the BBB presumably in the luminal membrane predisposing them as transporters for uptake into the brain (Gao et al., 2000; Bronger et al., 2005; Lee et al., 2005). In addition, OATP1A2 and OATP2B1 are also expressed in other CNS cell types. While both transporters have been identified in amacrine neurons of the retina, OATP1A2 was additionally found in hippocampal pyramidal and granule cells (Gao et al., 2015). Besides DHEAS, PregS levels in the brain may also be influenced by uptake transporters in the BBB, even though plasma concentrations of PregS were up to two orders of magnitude below the DHEAS levels (Sanchez-Guijo et al., 2015). Like for DHEAS, OATP-transporters are interesting candidates in this context. While PregS significantly inhibits OATP2B1 function (St Pierre et al., 2002; Grube et al., 2006) first reports indicated no direct transport of PregS by this transporter (Pizzagalli et al., 2003; Grube et al., 2006). Interestingly, OATP2B1-mediated transport of E_1_-3-S and DHEAS was stimulated by steroid hormones like progesterone (Grube et al., 2006; Koenen et al., 2012). Under these conditions, PregS was also transported by OATP2B1 (Grube et al., 2006). PregS transport by other OATPs has not been studied so far. With regard to the CP only limited information is available about OATP expression and function in humans. In a recent LC-MS/MS-based study examining transporter protein expression in this structure, only OATP3A1 was detected, while OATP1A2 and OATP1C1 were below the detection limit and OATP2B1 was not analyzed (Ji et al., 2012). This finding was quite surprising, since in animal models several OATPs have been detected in the CP and shown to be involved in the neurosteroid transport into the liquor (Asaba et al., 2000; Choudhuri et al., 2003; Ji et al., 2012). Due to the limited information available, the significance of OATP3A1 in this context cannot be conclusively assessed. However, a transport of E_1_-3-S has also been shown for the OATP3A1 and two splice variants of the transporter are selectively expressed in the apical and basal membrane of the ependymal cells of the CP (Tamai et al., 2000; Huber et al., 2007).

### Other Organic Anion Transporters (OSTα-OSTβ, OATs)

Besides OATPs, an interaction of neurosteroids (mainly DHEAS and PregS) with several further organic anion transporters has been reported. For example, both sulfated neurosteroids have been shown to be transported with high affinity by the organic solute transporter OSTα-OSTβ [K_m_: 1.5 μM (DHEAS) and 6.9 μM (PregS)] (Fang et al., 2010). The heterodimer OSTα-OSTβ is a relatively new member of the SLC family (SLC51) and encoded by two genes (SLC51A and SLC51B). Like OATPs, the transport mechanism is facilitated diffusion; therefore OSTα-OSTβ-mediated transport is dependent on the electrochemical gradient of its substrates (Ballatori et al., 2013). In the human brain, the transporter is expressed in Purkinje cells and hippocampal neurons (Fang et al., 2010). Both regions are well known for their function in the process of learning and memory, and hippocampal neurons have been suggested as a target site for PregS action (Akwa et al., 2001).

A third group of SLC transporters involved in the CNS distribution of sulfated neurosteroids are the organic anion transporters (OATs), which are part of the SLC22A branch (Burckhardt, 2012). The pivotal role of these transporters is the excretion of water-soluble organic anions in the kidney. However, selected members are also present in other organs including the brain (Burckhardt, 2012). In the brain OAT3, is probably the most interesting member of this family. The transporter is expressed in the BBB as well as the CP (Alebouyeh et al., 2003; Kikuchi et al., 2003; Uchida et al., 2015). In a mouse model, OAT3 was characterized as the DHEAS transporter in part responsible for the DHEAS efflux across the BBB (Miyajima et al., 2011). Besides OAT3, mRNA expression of OAT1 and OAT2 has also been shown for the human brain (Lopez-Nieto et al., 1997; Alebouyeh et al., 2003; Cropp et al., 2008); however, protein data for these transporters are limited.

## Conclusion and Perspectives

The detailed functions of the described ABC and SLC transporters in brain are still poorly understood. Even data on the expression and localization, e.g., at the BBB are often controversial. Knock-out mice of the ABC and some SLC transporters are available, but have been mainly used to study the role of these transporters for brain penetration of certain drugs (Dallas et al., 2006; Chaves et al., 2014). These studies are in part hampered by overlapping substrate specificities of several transporters and with respect to neurosteroids by the fact of negligible levels of sulfated steroids in rodent brain (Liu et al., 2003; Liere et al., 2009). Furthermore, a number of functional genetic variants in several transporter genes are known (Bruhn and Cascorbi, 2014); however, their impact on neurosteroid transport is so far largely unknown. Variations in transporter function may affect concentrations and action of several neurosteroids in brain. Therefore, a better understanding of these processes is an important aspect also in the context of a possible therapeutic use of these compounds.

## Author Contributions

MG and GJ conceived and wrote the manuscript. PH designed **Figure 1** and revised the manuscript. All authors read and approved the manuscript for publication.

## Conflict of Interest Statement

The authors declare that the research was conducted in the absence of any commercial or financial relationships that could be construed as a potential conflict of interest.

## Abbreviations

ABC: ATP-binding cassette
BBB: blood–brain barrier
BCRP: breast cancer resistance protein
CP: choroid plexus
CSF: cerebrospinal fluid
DHEAS: dehydroepiandrosterone sulfate
E_1_-3-S: estrone-3-sulfate
GABA: γ-aminobutyric acid
MRP: multidrug resistance protein
NMDA: *N*-methyl-D-aspartate
OATP: organic anion transporting polypeptide
OST: organic solute transporter
PregS: pregnenolone sulfate
SULT: sulfotransferase
TRP: transient receptor potential

## References

[B1] Acaz-Fonseca E. Avila-Rodriguez M. Garcia-Segura L. M. Barreto G. E. (2016). Regulation of astroglia by gonadal steroid hormones under physiological and pathological conditions. *Prog. Neurobiol.* 144 5–26. 10.1016/j.pneurobio.2016.06.002 27283249

[B2] Akwa Y. Ladurelle N. Covey D. F. Baulieu E. E. (2001). The synthetic enantiomer of pregnenolone sulfate is very active on memory in rats and mice, even more so than its physiological neurosteroid counterpart: distinct mechanisms? *Proc. Natl. Acad. Sci. U.S.A.* 98 14033–14037. 10.1073/pnas.241503698 11717462 PMC61162

[B3] Alebouyeh M. Takeda M. Onozato M. L. Tojo A. Noshiro R. Hasannejad H. (2003). Expression of human organic anion transporters in the choroid plexus and their interactions with neurotransmitter metabolites. *J. Pharmacol. Sci.* 93 430–436. 10.1254/jphs.93.430 14737013

[B4] Arbo B. D. Bennetti F. Ribeiro M. F. (2016). Astrocytes as a target for neuroprotection: modulation by progesterone and dehydroepiandrosterone. *Prog. Neurobiol.* 144 27–47. 10.1016/j.pneurobio.2016.03.010 27048624

[B5] Aronica E. Gorter J. A. Redeker S. van Vliet E. A. Ramkema M. Scheffer G. L. (2005). Localization of breast cancer resistance protein (BCRP) in microvessel endothelium of human control and epileptic brain. *Epilepsia* 46 849–857. 10.1111/j.1528-1167.2005.66604.x 15946326

[B6] Asaba H. Hosoya K. Takanaga H. Ohtsuki S. Tamura E. Takizawa T. Terasaki T. (2000). Blood-brain barrier is involved in the efflux transport of a neuroactive steroid, dehydroepiandrosterone sulfate, via organic anion transporting polypeptide 2. *J. Neurochem.* 75 1907–1916. 10.1046/j.1471-4159.2000.0751907.x 11032880

[B7] Ballatori N. Christian W. V. Wheeler S. G. Hammond C. L. (2013). The heteromeric organic solute transporter, OSTalpha-OSTbeta/SLC51: a transporter for steroid-derived molecules. *Mol. Aspects Med.* 34 683–692. 10.1016/j.mam.2012.11.005 23506901 PMC3827772

[B8] Baulieu E. E. (1997). Neurosteroids: of the nervous system, by the nervous system, for the nervous system. *Recent Prog. Horm. Res.* 52 1–32. 9238846

[B9] Belleville-Rolland T. Sassi Y. Decouture B. Dreano E. Hulot J. S. Gaussem P. (2016). MRP4 (ABCC4) as a potential pharmacologic target for cardiovascular disease. *Pharmacol. Res.* 107 381–389. 10.1016/j.phrs.2016.04.002 27063943

[B10] Bortfeld M. Rius M. Konig J. Herold-Mende C. Nies A. T. Keppler D. (2006). Human multidrug resistance protein 8 (MRP8/ABCC11), an apical efflux pump for steroid sulfates, is an axonal protein of the CNS and peripheral nervous system. *Neuroscience* 137 1247–1257. 10.1016/j.neuroscience.2005.10.025 16359813

[B11] Bronger H. Konig J. Kopplow K. Steiner H. H. Ahmadi R. Herold-Mende C. (2005). ABCC drug efflux pumps and organic anion uptake transporters in human gliomas and the blood-tumor barrier. *Cancer Res.* 65 11419–11428. 10.1158/0008-5472.CAN-05-1271 16357150

[B12] Bruhn O. Cascorbi I. (2014). Polymorphisms of the drug transporters ABCB1, ABCG2, ABCC2 and ABCC3 and their impact on drug bioavailability and clinical relevance. *Expert Opin. Drug Metab. Toxicol.* 10 1337–1354. 10.1517/17425255.2014.952630 25162314

[B13] Burckhardt G. (2012). Drug transport by organic anion transporters (OATs). *Pharmacol. Ther.* 136 106–130. 10.1016/j.pharmthera.2012.07.010 22841915

[B14] Chaves C. Shawahna R. Jacob A. Scherrmann J. M. Decleves X. (2014). Human ABC transporters at blood-CNS interfaces as determinants of CNS drug penetration. *Curr. Pharm. Des.* 20 1450–1462. 10.2174/13816128113199990466 23789951

[B15] Chen Z. S. Guo Y. Belinsky M. G. Kotova E. Kruh G. D. (2005). Transport of bile acids, sulfated steroids, estradiol 17-beta-D-glucuronide, and leukotriene C4 by human multidrug resistance protein 8 (ABCC11). *Mol. Pharmacol.* 67 545–557. 10.1124/mol.104.007138 15537867

[B16] Chen Z. S. Lee K. Kruh G. D. (2001). Transport of cyclic nucleotides and estradiol 17-beta-D-glucuronide by multidrug resistance protein 4. Resistance to 6-mercaptopurine and 6-thioguanine. *J. Biol. Chem.* 276 33747–33754. 10.1074/jbc.M104833200 11447229

[B17] Chisari M. Eisenman L. N. Covey D. F. Mennerick S. Zorumski C. F. (2010). The sticky issue of neurosteroids and GABA(A) receptors. *Trends Neurosci.* 33 299–306. 10.1016/j.tins.2010.03.005 20409596 PMC2902671

[B18] Choudhuri S. Cherrington N. J. Li N. Klaassen C. D. (2003). Constitutive expression of various xenobiotic and endobiotic transporter mRNAs in the choroid plexus of rats. *Drug Metab. Dispos.* 31 1337–1345. 10.1124/dmd.31.11.1337 14570765

[B19] Cole S. P. Bhardwaj G. Gerlach J. H. Mackie J. E. Grant C. E. Almquist K. C. (1992). Overexpression of a transporter gene in a multidrug-resistant human lung cancer cell line. *Science* 258 1650–1654. 10.1126/science.1360704 1360704

[B20] Cooray H. C. Blackmore C. G. Maskell L. Barrand M. A. (2002). Localisation of breast cancer resistance protein in microvessel endothelium of human brain. *Neuroreport* 13 2059–2063. 10.1097/00001756-200211150-00014 12438926

[B21] Cropp C. D. Komori T. Shima J. E. Urban T. J. Yee S. W. More S. S. (2008). Organic anion transporter 2 (SLC22A7) is a facilitative transporter of cGMP. *Mol. Pharmacol.* 73 1151–1158. 10.1124/mol.107.043117 18216183 PMC2698938

[B22] Dallas S. Miller D. S. Bendayan R. (2006). Multidrug resistance-associated proteins: expression and function in the central nervous system. *Pharmacol. Rev.* 58 140–161. 10.1124/pr.58.2.3 16714484

[B23] de Peretti E. Mappus E. (1983). Pattern of plasma pregnenolone sulfate levels in humans from birth to adulthood. *J. Clin. Endocrinol. Metab.* 57 550–556. 10.1210/jcem-57-3-550 6308031

[B24] Doyle L. A. Yang W. Abruzzo L. V. Krogmann T. Gao Y. Rishi A. K. (1998). A multidrug resistance transporter from human MCF-7 breast cancer cells. *Proc. Natl. Acad. Sci. U.S.A.* 95 15665–15670. 10.1073/pnas.95.26.15665 9861027 PMC28101

[B25] Fang F. Christian W. V. Gorman S. G. Cui M. Huang J. Tieu K. (2010). Neurosteroid transport by the organic solute transporter OSTalpha-OSTbeta. *J. Neurochem.* 115 220–233. 10.1111/j.1471-4159.2010.06920.x 20649839 PMC2939961

[B26] Gao B. Hagenbuch B. Kullak-Ublick G. A. Benke D. Aguzzi A. Meier P. J. (2000). Organic anion-transporting polypeptides mediate transport of opioid peptides across blood-brain barrier. *J. Pharmacol. Exp. Ther.* 294 73–79. 10871297

[B27] Gao B. Vavricka S. R. Meier P. J. Stieger B. (2015). Differential cellular expression of organic anion transporting peptides OATP1A2 and OATP2B1 in the human retina and brain: implications for carrier-mediated transport of neuropeptides and neurosteriods in the CNS. *Pflugers Arch.* 467 1481–1493. 10.1007/s00424-014-1596-x 25132355

[B28] Gazzin S. Strazielle N. Schmitt C. Fevre-Montange M. Ostrow J. D. Tiribelli C. (2008). Differential expression of the multidrug resistance-related proteins ABCb1 and ABCc1 between blood-brain interfaces. *J. Comp. Neurol.* 510 497–507. 10.1002/cne.21808 18680196

[B29] Grube M. Kock K. Karner S. Reuther S. Ritter C. A. Jedlitschky G. (2006). Modification of OATP2B1-Mediated transport by steroid hormones. *Mol. Pharmacol.* 70 1735–1741. 10.1124/mol.106.026450 16908597

[B30] Grube M. Reuther S. Meyer Zu Schwabedissen H. Kock K. Draber K. (2007). Organic anion transporting polypeptide 2B1 and breast cancer resistance protein interact in the transepithelial transport of steroid sulfates in human placenta. *Drug Metab. Dispos.* 35 30–35. 10.1124/dmd.106.011411 17020956

[B31] Hagenbuch B. Stieger B. (2013). The SLCO (former SLC21) superfamily of transporters. *Mol. Aspects Med.* 34 396–412. 10.1016/j.mam.2012.10.009 23506880 PMC3602805

[B32] Haimeur A. Conseil G. Deeley R. G. Cole S. P. (2004). The MRP-related and BCRP/ABCG2 multidrug resistance proteins: biology, substrate specificity and regulation. *Curr. Drug Metab.* 5 21–53. 10.2174/1389200043489199 14965249

[B33] Harteneck C. (2013). Pregnenolone sulfate: from steroid metabolite to TRP channel ligand. *Molecules* 18 12012–12028. 10.3390/molecules181012012 24084011 PMC6270300

[B34] Hirrlinger J. Konig J. Keppler D. Lindenau J. Schulz J. B. Dringen R. (2001). The multidrug resistance protein MRP1 mediates the release of glutathione disulfide from rat astrocytes during oxidative stress. *J. Neurochem.* 76 627–636. 10.1046/j.1471-4159.2001.00101.x 11208926

[B35] Hojo Y. Higo S. Kawato S. Hatanaka Y. Ooishi Y. Murakami G. (2011). Hippocampal synthesis of sex steroids and corticosteroids: essential for modulation of synaptic plasticity. *Front. Endocrinol.* 2:43. 10.3389/fendo.2011.00043 22701110 PMC3356120

[B36] Hoshi Y. Uchida Y. Tachikawa M. Inoue T. Ohtsuki S. Terasaki T. (2013). Quantitative atlas of blood-brain barrier transporters, receptors, and tight junction proteins in rats and common marmoset. *J. Pharm. Sci.* 102 3343–3355. 10.1002/jps.23575 23650139

[B37] Huber R. D. Gao B. Sidler P. fandler M. A. Zhang-Fu W. Leuthold S. (2007). Characterization of two splice variants of human organic anion transporting polypeptide 3A1 isolated from human brain. *Am. J. Physiol. Cell Physiol.* 292 C795–C806. 10.1152/ajpcell.00597.2005 16971491

[B38] Huss W. J. Gray D. R. Greenberg N. M. Mohler J. L. Smith G. J. (2005). Breast cancer resistance protein-mediated efflux of androgen in putative benign and malignant prostate stem cells. *Cancer Res.* 65 6640–6650. 10.1158/0008-5472.CAN-04-2548 16061644

[B39] Imai Y. Asada S. Tsukahara S. Ishikawa E. Tsuruo T. Sugimoto Y. (2003). Breast cancer resistance protein exports sulfated estrogens but not free estrogens. *Mol. Pharmacol.* 64 610–618. 10.1124/mol.64.3.610 12920197

[B40] Ito K. Uchida Y. Ohtsuki S. Aizawa S. Kawakami H. Katsukura Y. (2011). Quantitative membrane protein expression at the blood-brain barrier of adult and younger cynomolgus monkeys. *J. Pharm. Sci.* 100 3939–3950. 10.1002/jps.22487 21254069

[B41] Jedlitschky G. Greinacher A. Kroemer H. K. (2012). Transporters in human platelets: physiologic function and impact for pharmacotherapy. *Blood* 119 3394–3402. 10.1182/blood-2011-09-336933 22337717

[B42] Jedlitschky G. Leier I. Buchholz U. Barnouin K. Kurz G. Keppler D. (1996). Transport of glutathione, glucuronate, and sulfate conjugates by the MRP gene-encoded conjugate export pump. *Cancer Res.* 56 988–994. 8640791

[B43] Ji C. Tschantz W. R. Pfeifer N. D. Ullah M. Sadagopan N. (2012). Development of a multiplex UPLC-MRM MS method for quantification of human membrane transport proteins OATP1B1, OATP1B3 and OATP2B1 in in vitro systems and tissues. *Anal. Chim. Acta* 717 67–76. 10.1016/j.aca.2011.12.005 22304817

[B44] Kancheva R. Hill M. Novak Z. Chrastina J. Kancheva L. Starka L. (2011). Neuroactive steroids in periphery and cerebrospinal fluid. *Neuroscience* 191 22–27. 10.1016/j.neuroscience.2011.05.054 21641969

[B45] Kancheva R. Hill M. Novak Z. Chrastina J. Velikova M. Kancheva L. (2010). Peripheral neuroactive steroids may be as good as the steroids in the cerebrospinal fluid for the diagnostics of CNS disturbances. *J. Steroid Biochem. Mol. Biol.* 119 35–44. 10.1016/j.jsbmb.2009.12.006 20036740

[B46] Keppler D. (2011). Multidrug resistance proteins (MRPs, ABCCs): importance for pathophysiology and drug therapy. *Handb. Exp. Pharmacol.* 201 299–323. 10.1007/978-3-642-14541-4_8 21103974

[B47] Kikuchi R. Kusuhara H. Sugiyama D. Sugiyama Y. (2003). Contribution of organic anion transporter 3 (Slc22a8) to the elimination of p-aminohippuric acid and benzylpenicillin across the blood-brain barrier. *J. Pharmacol. Exp. Ther.* 306 51–58. 10.1124/jpet.103.049197 12684544

[B48] Kilic E. Spudich A. Kilic U. Rentsch K. M. Vig R. Matter C. M. (2008). ABCC1: a gateway for pharmacological compounds to the ischaemic brain. *Brain* 131 2679–2689. 10.1093/brain/awn222 18796513

[B49] Koenen A. Kock K. Keiser M. Siegmund W. Kroemer H. K. Grube M. (2012). Steroid hormones specifically modify the activity of organic anion transporting polypeptides. *Eur. J. Pharm. Sci.* 47 774–780. 10.1016/j.ejps.2012.08.017 22982504

[B50] Kool M. de Haas M. Scheffer G. L. Scheper R. J. van Eijk M. J. Juijn J. A. (1997). Analysis of expression of cMOAT (MRP2), MRP3, MRP4, and MRP5, homologues of the multidrug resistance-associated protein gene (MRP1), in human cancer cell lines. *Cancer Res.* 57 3537–3547. 9270026

[B51] Kubo Y. Ohtsuki S. Uchida Y. Terasaki T. (2015). Quantitative determination of luminal and abluminal membrane distributions of transporters in porcine brain capillaries by plasma membrane fractionation and quantitative targeted proteomics. *J. Pharm. Sci.* 104 3060–3068. 10.1002/jps.24398 25703048

[B52] Kullak-Ublick G. A. Fisch T. Oswald M. Hagenbuch B. Meier P. J. Beuers U. (1998). Dehydroepiandrosterone sulfate (DHEAS): identification of a carrier protein in human liver and brain. *FEBS Lett.* 424 173–176. 10.1016/S0014-5793(98)00168-9 9539145

[B53] Labrie F. Belanger A. Cusan L. Gomez J. L. Candas B. (1997). Marked decline in serum concentrations of adrenal C19 sex steroid precursors and conjugated androgen metabolites during aging. *J. Clin. Endocrinol. Metab.* 82 2396–2402. 10.1210/jcem.82.8.4160 9253307

[B54] Lee W. Glaeser H. Smith L. H. Roberts R. L. Moeckel G. W. Gervasini G. (2005). Polymorphisms in human organic anion-transporting polypeptide 1A2 (OATP1A2): implications for altered drug disposition and central nervous system drug entry. *J. Biol. Chem.* 280 9610–9617. 10.1074/jbc.M411092200 15632119

[B55] Leggas M. Adachi M. Scheffer G. L. Sun D. Wielinga P. Du G. (2004). Mrp4 confers resistance to topotecan and protects the brain from chemotherapy. *Mol. Cell. Biol.* 24 7612–7621. 10.1128/MCB.24.17.7612-7621.2004 15314169 PMC506999

[B56] Liere P. Pianos A. Eychenne B. Cambourg A. Bodin K. Griffiths W. (2009). Analysis of pregnenolone and dehydroepiandrosterone in rodent brain: cholesterol autoxidation is the key. *J. Lipid Res.* 50 2430–2444. 10.1194/jlr.M900162-JLR200 19506304 PMC2781315

[B57] Liu S. Sjovall J. Griffiths W. J. (2003). Neurosteroids in rat brain: extraction, isolation, and analysis by nanoscale liquid chromatography-electrospray mass spectrometry. *Anal. Chem.* 75 5835–5846. 10.1021/ac0346297 14588024

[B58] Loe D. W. Almquist K. C. Deeley R. G. Cole S. P. (1996). Multidrug resistance protein (MRP)-mediated transport of leukotriene C4 and chemotherapeutic agents in membrane vesicles. Demonstration of glutathione-dependent vincristine transport. *J. Biol. Chem.* 271 9675–9682. 10.1074/jbc.271.16.9675 8621643

[B59] Lopez-Nieto C. E. You G. Bush K. T. Barros E. J. Beier D. R. Nigam S. K. (1997). Molecular cloning and characterization of NKT, a gene product related to the organic cation transporter family that is almost exclusively expressed in the kidney. *J. Biol. Chem.* 272 6471–6478. 10.1074/jbc.272.10.6471 9045672

[B60] Maninger N. Wolkowitz O. M. Reus V. I. Epel E. S. Mellon S. H. (2009). Neurobiological and neuropsychiatric effects of dehydroepiandrosterone (DHEA) and DHEA sulfate (DHEAS). *Front. Neuroendocrinol.* 30 65–91. 10.1016/j.yfrne.2008.11.002 19063914 PMC2725024

[B61] Miller D. S. Nobmann S. N. Gutmann H. Toeroek M. Drewe J. Fricker G. (2000). Xenobiotic transport across isolated brain microvessels studied by confocal microscopy. *Mol. Pharmacol.* 58 1357–1367. 10.1124/mol.58.6.1357 11093774

[B62] Miyajima M. Kusuhara H. Fujishima M. Adachi Y. Sugiyama Y. (2011). Organic anion transporter 3 mediates the efflux transport of an amphipathic organic anion, dehydroepiandrosterone sulfate, across the blood-brain barrier in mice. *Drug Metab. Dispos.* 39 814–819. 10.1124/dmd.110.036863 21325432

[B63] Miyake K. Mickley L. Litman T. Zhan Z. Robey R. Cristensen B. (1999). Molecular cloning of cDNAs which are highly overexpressed in mitoxantrone-resistant cells: demonstration of homology to ABC transport genes. *Cancer Res.* 59 8–13. 9892175

[B64] Monnet F. P. Mahe V. Robel P. Baulieu E. E. (1995). Neurosteroids, via sigma receptors, modulate the [3H]norepinephrine release evoked by N-methyl-D-aspartate in the rat hippocampus. *Proc. Natl. Acad. Sci. U.S.A.* 92 3774–3778. 10.1073/pnas.92.9.3774 7731982 PMC42044

[B65] Mori S. Takanaga H. Ohtsuki S. Deguchi T. Kang Y. S. Hosoya K. (2003). Rat organic anion transporter 3 (rOAT3) is responsible for brain-to-blood efflux of homovanillic acid at the abluminal membrane of brain capillary endothelial cells. *J. Cereb. Blood Flow Metab.* 23 432–440. 10.1097/01.WCB.0000050062.57184.75 12679720

[B66] Niehof M. Borlak J. (2009). Expression of HNF4alpha in the human and rat choroid plexus: implications for drug transport across the blood-cerebrospinal-fluid (CSF) barrier. *BMC Mol. Biol.* 10:68. 10.1186/1471-2199-10-68 19575803 PMC2713241

[B67] Nies A. T. Jedlitschky G. Konig J. Herold-Mende C. Steiner H. H. Schmitt H. P. (2004). Expression and immunolocalization of the multidrug resistance proteins, MRP1-MRP6 (ABCC1-ABCC6), in human brain. *Neuroscience* 129 349–360. 10.1016/j.neuroscience.2004.07.051 15501592

[B68] Nishimura M. Naito S. (2005). Tissue-specific mRNA expression profiles of human ATP-binding cassette and solute carrier transporter superfamilies. *Drug Metab. Pharmacokinet.* 20 452–477. 10.2133/dmpk.20.452 16415531

[B69] Nishizawa K. Nakanishi T. Tamai I. (2017). Comparative evaluation of dehydroepiandrosterone sulfate potential to predict hepatic organic anion transporting polypeptide transporter-based drug-drug interactions. *Drug Metab. Dispos.* 45 224–227. 10.1124/dmd.116.072355 27934638

[B70] Pizzagalli F. Hagenbuch B. Stieger B. Klenk U. Folkers G. Meier P. J. (2002). Identification of a novel human organic anion transporting polypeptide as a high affinity thyroxine transporter. *Mol. Endocrinol.* 16 2283–2296. 10.1210/me.2001-0309 12351693

[B71] Pizzagalli F. Varga Z. Huber R. D. Folkers G. Meier P. J. St Pierre M. V. (2003). Identification of steroid sulfate transport processes in the human mammary gland. *J. Clin. Endocrinol. Metab.* 88 3902–3912. 10.1210/jc.2003-030174 12915686

[B72] Porcu P. Barron A. M. Frye C. A. Walf A. A. Yang S. Y. He X. Y. (2016). Neurosteroidogenesis today: novel targets for neuroactive steroid synthesis and action and their relevance for translational research. *J. Neuroendocrinol.* 28:12351. 10.1111/jne.12351 26681259 PMC4769676

[B73] Qian Y. M. Song W. C. Cui H. Cole S. P. Deeley R. G. (2001). Glutathione stimulates sulfated estrogen transport by multidrug resistance protein 1. *J. Biol. Chem.* 276 6404–6411. 10.1074/jbc.M008251200 11102445

[B74] Rao V. V. Dahlheimer J. L. Bardgett M. E. Snyder A. Z. Finch R. A. Sartorelli A. C. (1999). Choroid plexus epithelial expression of MDR1 P glycoprotein and multidrug resistance-associated protein contribute to the blood-cerebrospinal-fluid drug-permeability barrier. *Proc. Natl. Acad. Sci. U.S.A.* 96 3900–3905. 10.1073/pnas.96.7.3900 10097135 PMC22392

[B75] Reddy D. S. (2010). Neurosteroids: endogenous role in the human brain and therapeutic potentials. *Prog. Brain Res.* 18 113–137. 10.1016/B978-0-444-53630-3.00008-7 21094889 PMC3139029

[B76] Ritter C. A. Jedlitschky G. Meyer Zu Schwabedissen H. Grube M. Kock K. Kroemer H. K. (2005). Cellular export of drugs and signaling molecules by the ATP-binding cassette transporters MRP4 (ABCC4) and MRP5 (ABCC5). *Drug Metab. Rev.* 37 253–278. 10.1081/DMR-200047984 15747503

[B77] Roberts L. M. Black D. S. Raman C. Woodford K. Zhou M. Haggerty J. E. (2008a). Subcellular localization of transporters along the rat blood-brain barrier and blood-cerebral-spinal fluid barrier by in vivo biotinylation. *Neuroscience* 155 423–438. 10.1016/j.neuroscience.2008.06.015 18619525

[B78] Roberts L. M. Woodford K. Zhou M. Black D. S. Haggerty J. E. Tate E. H. (2008b). Expression of the thyroid hormone transporters monocarboxylate transporter-8 (SLC16A2) and organic ion transporter-14 (SLCO1C1) at the blood-brain barrier. *Endocrinology* 149 6251–6261. 10.1210/en.2008-0378 18687783

[B79] Salman E. D. Faye-Petersen O. Falany C. N. (2011). Hydroxysteroid sulfotransferase 2B1b expression and localization in normal human brain. *Horm. Mol. Biol. Clin. Investig.* 8 445–454. 10.1515/HMBCI.2011.117 24683427 PMC3966311

[B80] Sanchez-Guijo A. Oji V. Hartmann M. F. Traupe H. Wudy S. A. (2015). Simultaneous quantification of cholesterol sulfate, androgen sulfates, and progestagen sulfates in human serum by LC-MS/MS. *J. Lipid Res.* 56 1843–1851. 10.1194/jlr.D061499 26239050 PMC4548788

[B81] Seljeset S. Laverty D. Smart T. G. (2015). Inhibitory neurosteroids and the GABAA receptor. *Adv. Pharmacol.* 72 165–187. 10.1016/bs.apha.2014.10.006 25600370

[B82] Shawahna R. Uchida Y. Decleves X. Ohtsuki S. Yousif S. Dauchy S. (2011). Transcriptomic and quantitative proteomic analysis of transporters and drug metabolizing enzymes in freshly isolated human brain microvessels. *Mol. Pharm.* 8 1332–1341. 10.1021/mp200129p 21707071

[B83] Spiegl-Kreinecker S. Buchroithner J. Elbling L. Steiner E. Wurm G. Bodenteich A. (2002). Expression and functional activity of the ABC-transporter proteins P-glycoprotein and multidrug-resistance protein 1 in human brain tumor cells and astrocytes. *J. Neurooncol.* 57 27–36. 10.1023/A:1015735815111 12125964

[B84] St Pierre M. V. Hagenbuch B. Ugele B. Meier P. J. Stallmach T. (2002). Characterization of an organic anion-transporting polypeptide (OATP-B) in human placenta. *J. Clin. Endocrinol. Metab.* 87 1856–1863. 10.1210/jcem.87.4.8431 11932330

[B85] Suzuki M. Suzuki H. Sugimoto Y. Sugiyama Y. (2003). ABCG2 transports sulfated conjugates of steroids and xenobiotics. *J. Biol. Chem.* 278 22644–22649. 10.1074/jbc.M212399200 12682043

[B86] Tamai I. Nezu J. Uchino H. Sai Y. Oku A. Shimane M. (2000). Molecular identification and characterization of novel members of the human organic anion transporter (OATP) family. *Biochem. Biophys. Res. Commun.* 273 251–260. 10.1006/bbrc.2000.2922 10873595

[B87] Uchida Y. Ohtsuki S. Katsukura Y. Ikeda C. Suzuki T. Kamiie J. (2011). Quantitative targeted absolute proteomics of human blood-brain barrier transporters and receptors. *J. Neurochem.* 117 333–345. 10.1111/j.1471-4159.2011.07208.x 21291474

[B88] Uchida Y. Zhang Z. Tachikawa M. Terasaki T. (2015). Quantitative targeted absolute proteomics of rat blood-cerebrospinal fluid barrier transporters: comparison with a human specimen. *J. Neurochem.* 134 1104–1115. 10.1111/jnc.13147 25951748

[B89] Wang D. Y. Bulbrook R. D. (1969). The binding of steroids to plasma proteins in normal women and women with breast cancer. *Eur. J. Cancer* 5 247–253. 10.1016/0014-2964(69)90074-7 5786067

[B90] Warren M. S. Zerangue N. Woodford K. Roberts L. M. Tate E. H. Feng B. (2009). Comparative gene expression profiles of ABC transporters in brain microvessel endothelial cells and brain in five species including human. *Pharmacol. Res.* 59 404–413. 10.1016/j.phrs.2009.02.007 19429473

[B91] Watanabe M. Watanabe T. Yabuki M. Tamai I. (2015). Dehydroepiandrosterone sulfate, a useful endogenous probe for evaluation of drug-drug interaction on hepatic organic anion transporting polypeptide (OATP) in cynomolgus monkeys. *Drug Metab. Pharmacokinet.* 30 198–204. 10.1016/j.dmpk.2014.12.009 25989893

[B92] Weill-Engerer S. David J. P. Sazdovitch V. Liere P. Eychenne B. Pianos A. (2002). Neurosteroid quantification in human brain regions: comparison between Alzheimer’s and nondemented patients. *J. Clin. Endocrinol. Metab.* 87 5138–5143. 10.1210/jc.2002-020878 12414884

[B93] Wijnholds J. deLange E. C. Scheffer G. L. van den Berg D. J. Mol C. A. van D. V. (2000). Multidrug resistance protein 1 protects the choroid plexus epithelium and contributes to the blood-cerebrospinal fluid barrier. *J. Clin. Invest.* 105 279–285. 10.1172/JCI8267 10675353 PMC377447

[B94] Wu F. S. Gibbs T. T. Farb D. H. (1991). Pregnenolone sulfate: a positive allosteric modulator at the N-methyl-D-aspartate receptor. *Mol. Pharmacol.* 40 333–336. 1654510

[B95] Zelcer N. Reid G. Wielinga P. Kuil A. van D. H. I. Schuetz J. D. (2003). Steroid and bile acid conjugates are substrates of human multidrug-resistance protein (MRP) 4 (ATP-binding cassette C4). *Biochem. J.* 371 361–367. 10.1042/bj20021886 12523936 PMC1223295

[B96] Zhang Y. Schuetz J. D. Elmquist W. F. Miller D. W. (2004). Plasma membrane localization of multidrug resistance-associated protein homologs in brain capillary endothelial cells. *J. Pharmacol. Exp. Ther.* 311 449–455. 10.1124/jpet.104.068528 15218051

